# Effect of Educational Interventions to Reduce Readmissions due to Heart Failure Decompensation in Adults: a Systematic Review and Meta-analysis

**DOI:** 10.17533/udea.iee.v39n2e05

**Published:** 2021-06-15

**Authors:** Wilson Cañon-Montañez, Tatiana Duque-Cartagena, Alba Luz Rodríguez-Acelas

**Affiliations:** 1 RN, Ph.D. Associate Professor. Email: wilson.canon@udea.edu.co. Corresponding author. Universidad de Antioquia Colombia wilson.canon@udea.edu.co; 2 Undergraduate nursing student, young researcher. Universidad de Antioquia. Medellín, Colombia Email: tatiana.duquec@udea.edu.co Universidad de Antioquia Universidad de Antioquia Medellín Colombia tatiana.duquec@udea.edu.co; 3 RN, Ph.D. Associate Professor. Universidad de Antioquia. Medellín, ColombiaEmail: aluz.rodriguez@udea.edu.co Universidad de Antioquia Universidad de Antioquia Medellín Colombia aluz.rodriguez@udea.edu.co; 4 Faculty of Nursing, Universidad de Antioquia. Medellín, Colombia. Universidad de Antioquia Universidad de Antioquia Medellín Colombia

**Keywords:** heart failure, patient readmission, patient education as topic, self-care, systematic review., insuficiencia cardiaca, readmisión del paciente, educación del paciente como asunto, autocuidado, revisión sistemática., insuficiência cardíaca, readmissão do paciente, educação de pacientes como assunto, autocuidado, revisão sistemática.

## Abstract

**Objective.:**

To estimate the combined effect of educational interventions (EI) on decreased readmissions and time of hospital stay in adults with heart failure, compared with usual care.

**Methods.:**

Systematic review (SR) and meta-analysis (MA) of randomized controlled trials that followed the recommendations of the PRISMA statement. The protocol was registered on PROSPERO (CRD42019139321). Searches were made from inception until July 2019 in the databases of PubMed/Medline, Embase, Cochrane CENTRAL, Lilacs, Web of Science, and Scopus. The MA was conducted through the random effects model. The effect measure used for the dichotomous outcomes was relative risk (RR) and for continuous outcomes the mean difference (MD) was used, with 95% confidence intervals (CI). Heterogeneity was evaluated through the inconsistency statistic (I^2^).

**Results.:**

Of 2369 studies identified, 45 were included in the SR and 43 in the MA. The MA of studies with follow-up at six months showed a decrease in readmissions of 30% (RR: 0.70; 95% CI: 0.58 to 0.84; I^2^: 0%) and the 12-month follow-up evidenced a reduction of 33% (RR: 0.67; 95% CI: 0.58 to 0.76; I^2^: 52%); both analyses in favor of the EI group. Regarding the time of hospital stay, a reduction was found of approximately two days in patients who received the EI (MD: -1.98; 95% CI: -3.27 to -0.69; I^2^: 7%).

**Conclusion.:**

The findings support the benefits of EI to reduce readmissions and days of hospital stay in adult patients with heart failure.

## Resumen

**Objetivo.:**

Estimar el efecto combinado de las intervenciones educativas (IE) en la disminución de readmisiones y tiempo de estancia hospitalaria en adultos con falla cardiaca comparado con el cuidado usual.

**Métodos.:**

Revisión Sistemática (RS) y meta-análisis (MA) de ensayos clínicos aleatorizados que siguieron las recomendaciones de la declaración PRISMA. El protocolo se registró en PROSPERO (CRD42019139321). Se realizaron búsquedas desde el inicio hasta julio de 2019, en las bases de datos PubMed/Medline, Embase, Cochrane CENTRAL, Lilacs, Web of Science y Scopus. El MA se realizó mediante modelo de efectos aleatorios. La medida de efecto utilizada para los desenlaces dicotómicos fue el riesgo relativo (RR) y para desenlaces continuos se usó la diferencia de medias (DM), con sus intervalos de confianza (IC) del 95%. La heterogeneidad se evaluó mediante el estadístico de inconsistencia (I^2^).

**Resultados.:**

De 2369 estudios identificados, 45 se incluyeron en la RS y 43 en el MA. El MA de estudios con seguimiento a seis meses mostró una disminución en las readmisiones de 30% (RR: 0.70; IC 95%: 0.58 a 0.84; I^2^: 0%) y el seguimiento a doce meses evidenció una reducción de 33% (RR: 0.67; IC 95%: 0.58 a 0.76; I^2^: 52%), ambos análisis a favor del grupo de IE. Referente al tiempo de estancia hospitalaria, se encontró una reducción de aproximadamente dos días en los pacientes que recibieron las IE (DM: -1.98; IC 95%: -3.27 a -0.69; I^2^: 7%).

**Conclusión.:**

Los hallazgos soportan los beneficios de las IE para la disminución de readmisiones y días de estancia hospitalaria en pacientes adultos con falla cardiaca.

## Resumo

**Objetivo.:**

Estimar o efeito combinado de intervenções educacionais (IE) na redução de readmissões e tempo de internação em adultos com insuficiência cardíaca, em comparação com o cuidado usual.

**Métodos.:**

Revisão sistemática (RS) e meta-análise (MA) de ensaios clínicos randomizados que seguiu as recomendações da declaração PRISMA. O protocolo foi registrado no PROSPERO (CRD42019139321). Foram realizadas buscas desde o início até julho de 2019, nas bases de dados PubMed/Medline, Embase, Cochrane CENTRAL, Lilacs, Web of Science e Scopus. A MA foi realizada usando um modelo de efeitos aleatórios. A medida de efeito utilizada para desfechos dicotômicos foi o risco relativo (RR) e para desfechos contínuos foi usada a diferença de médias (DM), com seus intervalos de confiança (IC) de 95%. A heterogeneidade foi avaliada por meio da estatística de inconsistência (I^2^).

**Resultados.:**

De 2369 estudos identificados, 45 foram incluídos na RS e 43 na MA. A MA dos estudos com seguimento de seis meses mostrou uma diminuição nas readmissões de 30% (RR: 0.70; IC 95%: 0.58 a 0.84; I^2^: 0%) e o seguimento de doze meses mostrou uma redução de 33 % (RR: 0.67; IC 95%: 0.58 a 0.76; I^2^: 52%), ambas as análises em favor do grupo de IE**.** Em relação ao tempo de internação, foi observada uma redução de aproximadamente dois dias nos pacientes que receberam as IE (DM: -1.98; IC 95%: -3.27 a -0.69; I^2^: 7%).

**Conclusão.:**

Os achados evidenciam os benefícios das IE para a redução de readmissões e dias de internação em pacientes adultos com insuficiência cardíaca.

## Introduction

Heart failure (HF) is part of the group of cardiovascular diseases. Defining this disease is complex, given that it involves different processes and its etiology is also varied, which is why it is referred to as a “syndrome”. Simply stated, it may be understood as “state in which the heart is not capable to pump the amount of blood necessary to fulfil the needs of the organism”.(1) Moreover, due to its high morbidity and mortality figures,(2,3) currently, HF is considered a public health problem, besides implying a high cost for governments and health systems. Evidence shows that the prevalence of HF increases gradually with age and it is estimated to affect 10% of elderly adults, becoming the first cause of hospitalization in this population.(4) 

In relation with the socioeconomic burden due to HF, some European and South American countries show high costs for health services;(3,5,6) which has become a great concern for the governments and health institutions. Another one of the serious problems of HF is the increase of readmissions of patients due to the decompensation of the disease.(4) Within this context, over time, specialized units have been created with programs of multidisciplinary approach for the integral management of patients with HF.(3) Among these programs, education of patients is crucial to improve the clinical outcomes of patients. Health education is one of the professional roles of nursing. Nurses must have the ability to evaluate the patients’ individual needs for education and be able to improve their self-care practices that contribute to the reduction of readmissions.(2) Educational interventions can vary in their intensity, methodology, or strategy. The effect sought with these interventions is to achieve a greater number of patients with HF aware of their disease and of the importance of self-care habits for their health. This, in turn, favors better control of the disease and reduction of the different complications and costs associated with HF.(5,6)

Due to the aforementioned, up-to-date syntheses are required of the literature that evidences the effect the educational interventions have on reducing readmissions due to decompensation of the HF syndrome. Although primary studies exist to address this problem, it is important to group systematically every evidence to permit greater comprehension of the phenomenon and generate new results that contribute to the recovery of individuals who endure this disease. Hence, the objective of this study was to estimate the combined effect of the educational interventions on reducing hospital readmissions and time of hospital stay in adults with HF, compared with usual care.

## Methods

Design and registry of the protocol. This was a systematic review (SR) and meta-analysis (MA) of randomized controlled trials (RCTs) that followed the recommendations of the PRISMA (Preferred Reporting Items for Systematic Reviews and Meta-Analyses) statement (7) and of the Cochrane Handbook (8) for SR of intervention studies. The protocol was registered in the International Prospective Register of Systematic Reviews (PROSPERO) with code CRD42019139321.

Source of data and search strategy. The information was collected from the following electronic databases: PubMed/Medline, Embase, Lilacs, Cochrane CENTRAL, Scopus, and Web of Science. Searches were made from inception until July 2019, using MeSH terms and entry terms for PubMed/Medline, emtree terms for Embase and descriptors for the other databases. Likewise, the following filters were used for the search strategy: randomized controlled trials, studies in humans and English, Portuguese, Spanish languages. To identify additional studies, search was made in other sources that included the review of references of the studies included, SR published, and the network of primary registries of RCTs recognized by the World Health Organization.

The following search strategy was used for PubMed/Medline: *((Heart failure[MeSH Terms]) OR (Cardiac Failure)) OR (Heart Decompensation)) OR (Decompensation, Heart)) OR (Congestive heart failure)) OR (Heart Failure, Congestive)) )) AND (Knowledge[MeSH Terms])) AND (Self-care[MeSH Terms]))) OR (Care, Self)) OR (Self-care behaviors[MeSH Terms])) OR (Self-management[MeSH Terms])) OR (Management, Self)) OR (Self-efficacy[MeSH Terms])) OR (Efficacy, Self)) OR (Self Concept[MeSH Terms])) OR (Self-confidence)) OR (Confidence, Self)) AND (Education[MeSH Terms])) AND (Patient education[MeSH Terms]) ) ) OR (Education, Patient)) ) OR (Education of Patients) ) AND (Education, nursing [MeSH Terms])) ) OR (Nursing Education)) OR (Educations, Nursing)) OR (Nursing Educations)) AND (Health education[MeSH Terms])) OR (Education, Health)) AND (Standard of Care)*

Eligibility criteria of the studies. This SR and MA included experimental studies or CCT-type intervention studies. The following PICO (population, intervention, comparator, outcomes) research question was used to consider the eligibility of the studies, P: adult patients with HF in any stage of the disease; I: educational interventions; C: usual or standard care, and O: reduced readmissions and time of hospital stay due to decompensation of the HF.

Data extraction. Identification and selection of the studies was performed independently by two reviewers, who were young undergraduate researchers with prior training and certification in SR and MA. Disagreements were solved through the intervention of a third reviewer, senior researcher with PhD formation and experience in SR and MA. Articles duplicated in several databases were considered only once. The Mendeley reference manager was used to store references and eliminate duplicate studies.

Outcomes. The principal outcome was the decrease of hospital readmissions due to decompensation of the HF and the secondary outcome was the decrease of days of hospital stay.

Evaluation of the risk of study bias. The risk of bias (RoB 1) tool from the Cochrane Collaboration (9) was used to evaluate the risk of bias in RCTs*.* The following parameters were evaluated: random sequence generation and allocation concealment, blinding of participants and personnel, blinding of outcome assessment, incomplete outcome data, selective reporting of results and other sources of bias.

Data analysis. Estimation of the grouped effect was conducted with the Review Manager (RevMan 5.4) software from the Cochrane Collaboration. The dichotomous results are presented and compared by using relative risk (RR) through the Mantel-Haenszel method and for continuous results the mean difference (MD) is presented through the inverse-variance weighted; both with their respective 95% confidence intervals (CI). Likewise, to quantify the heterogeneity of the studies included, the inconsistency (I^2^) statistic was used and the graphic presentation of the MA results used the forest plot. To evaluate publication bias or bias due to missing results, the Stata 16.0 software was used, through the Egger test and the funnel plot.

## Results

### Identification and selection of the studies

**Figure 1:**
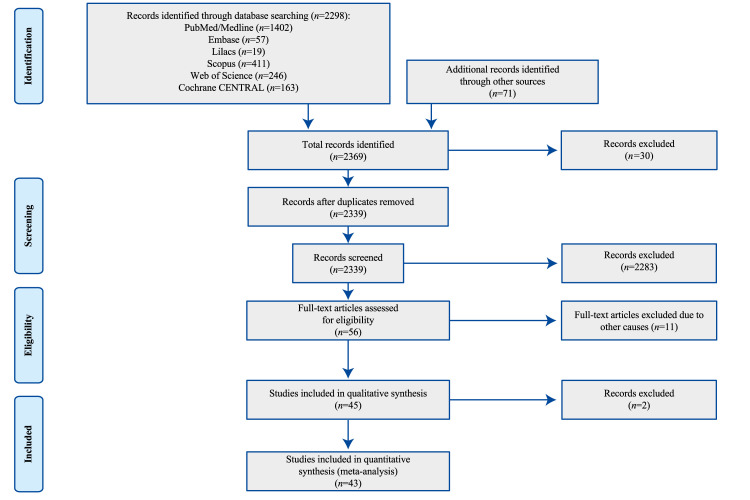
PRISMA flow diagram for the studies selection

The work identified 2369 studies, of which 45 studies were included in the SR and data from 43 studies were included in the MA. Two studies were excluded from the MA because the data on readmissions corresponded to follow-up times different from the other studies and, hence, it was not possible to meta-analyze. The flow diagram for the selection and exclusion of studies is shown in Figure 1.

### Characteristics of studies included

The general description of the studies is shown in Table 1, which contains the author, year of publication, country, a brief description of the intervention, time of follow-up, and most relevant results for the research.

**Table 1:** Characteristics of the included studies

First author, year	Country	Sample size	Intervention group	Follow-up	Control group	Main outcomes
Aldamiz-Echevarría *et al*., 2007 (10)	Spain	279	Educational program on basic data of HF and its treatment.	3, 6 and 12 months	Standard care	Readmissions* at 12 months
Aldamiz-Echevarría *et al*., 2007 (10)	Spain	279	Educational program on basic data of HF and its treatment.	3, 6 and 12 months	Standard care	Intervention: 55
Aldamiz-Echevarría *et al*., 2007 (10)	Spain	279	Educational program on basic data of HF and its treatment.	3, 6 and 12 months	Standard care	Control: 57
Aldamiz-Echevarría *et al*., 2007 (10)	Spain	279	Educational program on basic data of HF and its treatment.	3, 6 and 12 months	Standard care	Days of hospital stay+
Aldamiz-Echevarría *et al*., 2007 (10)	Spain	279	Educational program on basic data of HF and its treatment.	3, 6 and 12 months	Standard care	Intervention: 8.5 (6.4)
Aldamiz-Echevarría *et al*., 2007 (10)	Spain	279	Educational program on basic data of HF and its treatment.	3, 6 and 12 months	Standard care	Control: 8.4 (11.6)
Atienza *et al*., 2004 (11)	Spain	338	Education before discharge on knowledge of the disease and its management. Home visits.	12 months	Standard care	Readmissions* at 12 months Intervention: 61 Control: 122
Atienza *et al*., 2004 (11)	Spain	338	Education before discharge on knowledge of the disease and its management. Home visits.	12 months	Standard care	Intervention: 61
Atienza *et al*., 2004 (11)	Spain	338	Education before discharge on knowledge of the disease and its management. Home visits.	12 months	Standard care	Control: 122
Blue *et al*., 2001 (12)	Scotland	165	Education through home visits and telecare on knowledge and treatment of HF. Educational brochure. Instruments for self-monitoring.	12 months	Standard care	Readmissions* at 12 months
Blue *et al*., 2001 (12)	Scotland	165	Education through home visits and telecare on knowledge and treatment of HF. Educational brochure. Instruments for self-monitoring.	12 months	Standard care	Intervention: 12
Blue *et al*., 2001 (12)	Scotland	165	Education through home visits and telecare on knowledge and treatment of HF. Educational brochure. Instruments for self-monitoring.	12 months	Standard care	Control: 26
Blue *et al*., 2001 (12)	Scotland	165	Education through home visits and telecare on knowledge and treatment of HF. Educational brochure. Instruments for self-monitoring.	12 months	Standard care	Days of hospital stay+
Blue *et al*., 2001 (12)	Scotland	165	Education through home visits and telecare on knowledge and treatment of HF. Educational brochure. Instruments for self-monitoring.	12 months	Standard care	Intervention: 3.43 (12.2)
Blue *et al*., 2001 (12)	Scotland	165	Education through home visits and telecare on knowledge and treatment of HF. Educational brochure. Instruments for self-monitoring.	12 months	Standard care	Control: 7.46 (16.6)
Boyde *et al*., 2018 (13)	United States	200	Education prior to discharge on HF. Brochure and video.	1, 3 and 12 months	Standard care	Readmissions* at 3 months
Boyde *et al*., 2018 (13)	United States	200	Education prior to discharge on HF. Brochure and video.	1, 3 and 12 months	Standard care	Intervention: 8
Boyde *et al*., 2018 (13)	United States	200	Education prior to discharge on HF. Brochure and video.	1, 3 and 12 months	Standard care	Control: 10
Boyde *et al*., 2018 (13)	United States	200	Education prior to discharge on HF. Brochure and video.	1, 3 and 12 months	Standard care	Readmissions* at 12 months
Boyde *et al*., 2018 (13)	United States	200	Education prior to discharge on HF. Brochure and video.	1, 3 and 12 months	Standard care	Intervention: 8
Boyde *et al*., 2018 (13)	United States	200	Education prior to discharge on HF. Brochure and video.	1, 3 and 12 months	Standard care	Control: 14
Brian *et al*., 2009 (14)	United States	749	Education on HF. Brochure and telephone follow-up.	1 month	Standard care	Readmissions* at 1 month
Brian *et al*., 2009 (14)	United States	749	Education on HF. Brochure and telephone follow-up.	1 month	Standard care	Intervention: 55
Brian *et al*., 2009 (14)	United States	749	Education on HF. Brochure and telephone follow-up.	1 month	Standard care	Control:76
Brotons *et al*., 2009 (15)	Spain	283	Pre-discharge education on HF with brochure. Home visits for one year. Phone follow-up every 15 days.	12 months	Standard care	Readmissions* at 12 months
Brotons *et al*., 2009 (15)	Spain	283	Pre-discharge education on HF with brochure. Home visits for one year. Phone follow-up every 15 days.	12 months	Standard care	Intervention: 52
Brotons *et al*., 2009 (15)	Spain	283	Pre-discharge education on HF with brochure. Home visits for one year. Phone follow-up every 15 days.	12 months	Standard care	Control: 62
Cañon-Montañez *et al*., 2013 (16)	Colombia	116	Education on HF and its management. Face to face and phone education.	1 and 2 months	Standard care (phone call)	Readmissions* at 2 months
Cañon-Montañez *et al*., 2013 (16)	Colombia	116	Education on HF and its management. Face to face and phone education.	1 and 2 months	Standard care (phone call)	Intervention: 11
Cañon-Montañez *et al*., 2013 (16)	Colombia	116	Education on HF and its management. Face to face and phone education.	1 and 2 months	Standard care (phone call)	Control: 5
Cañon-Montañez *et al*., 2013 (16)	Colombia	116	Education on HF and its management. Face to face and phone education.	1 and 2 months	Standard care (phone call)	Days of hospital stay at 2 months
Cañon-Montañez *et al*., 2013 (16)	Colombia	116	Education on HF and its management. Face to face and phone education.	1 and 2 months	Standard care (phone call)	Intervention: 6.27 (5.93)
Cañon-Montañez *et al*., 2013 (16)	Colombia	116	Education on HF and its management. Face to face and phone education.	1 and 2 months	Standard care (phone call)	Control: 11 (11)
Cui *et al*., 2019 (17)	China	96	Structured education in HF for one hour upon admission, and one hour before discharge. Phone or face-to-face consultation every 4 weeks.	12 months	Standard care	Readmissions* at 12 months
Cui *et al*., 2019 (17)	China	96	Structured education in HF for one hour upon admission, and one hour before discharge. Phone or face-to-face consultation every 4 weeks.	12 months	Standard care	Intervention: 5
Cui *et al*., 2019 (17)	China	96	Structured education in HF for one hour upon admission, and one hour before discharge. Phone or face-to-face consultation every 4 weeks.	12 months	Standard care	Control: 13
Davis *et al*., 2012 (18)	United States	125	Education during hospitalization. Phone call after discharge. Video with recorded sessions. Supplies to aid self-care.	1 month	Standard care	Readmissions* at 1 month
Davis *et al*., 2012 (18)	United States	125	Education during hospitalization. Phone call after discharge. Video with recorded sessions. Supplies to aid self-care.	1 month	Standard care	Intervention: 14
Davis *et al*., 2012 (18)	United States	125	Education during hospitalization. Phone call after discharge. Video with recorded sessions. Supplies to aid self-care.	1 month	Standard care	Control: 12
De Souza *et al.,* 2014 (19)	Brazil	252	Home visits to educate on HF. Phone calls to reinforce education.	6 months	Standard care	Readmissions* at 6 months
De Souza *et al.,* 2014 (19)	Brazil	252	Home visits to educate on HF. Phone calls to reinforce education.	6 months	Standard care	Intervention: 20
De Souza *et al.,* 2014 (19)	Brazil	252	Home visits to educate on HF. Phone calls to reinforce education.	6 months	Standard care	Control: 30
DeBusk *et al*., 2004 (20)	United States	462	Education with a videotape. Telephone counseling and printed educational materials.	12 months	Standard care	Readmissions* at 12 months
DeBusk *et al*., 2004 (20)	United States	462	Education with a videotape. Telephone counseling and printed educational materials.	12 months	Standard care	Intervention: 76
DeBusk *et al*., 2004 (20)	United States	462	Education with a videotape. Telephone counseling and printed educational materials.	12 months	Standard care	Control: 86
Delaney *et al*., 2013 (21)	United States	100	Telemonitoring. Brochure with information on HF and its management.	3 months	Standard care	Readmissions* at 3 months
Delaney *et al*., 2013 (21)	United States	100	Telemonitoring. Brochure with information on HF and its management.	3 months	Standard care	Intervention: 3
Delaney *et al*., 2013 (21)	United States	100	Telemonitoring. Brochure with information on HF and its management.	3 months	Standard care	Control: 7
Dewalt *et al*., 2006 (22)	United States	127	Education on HF and warning signs. Phone calls for reinforcement of the education. Educational brochure.	6 and 12 months	Standard care	Readmissions* at 12 months
Dewalt *et al*., 2006 (22)	United States	127	Education on HF and warning signs. Phone calls for reinforcement of the education. Educational brochure.	6 and 12 months	Standard care	Intervention: 18
Dewalt *et al*., 2006 (22)	United States	127	Education on HF and warning signs. Phone calls for reinforcement of the education. Educational brochure.	6 and 12 months	Standard care	Control: 20
Domingues *et al*., 2011 (23)	Brazil	120	Phone calls after hospital discharge to educate and evaluate signs of decompensation of HF.	3 months	Standard care	Readmissions* at 3 months
Domingues *et al*., 2011 (23)	Brazil	120	Phone calls after hospital discharge to educate and evaluate signs of decompensation of HF.	3 months	Standard care	Intervention: 20
Domingues *et al*., 2011 (23)	Brazil	120	Phone calls after hospital discharge to educate and evaluate signs of decompensation of HF.	3 months	Standard care	Control: 23
Domingues *et al*., 2011 (23)	Brazil	120	Phone calls after hospital discharge to educate and evaluate signs of decompensation of HF.	3 months	Standard care	Readmissions* at 12 months
Domingues *et al*., 2011 (23)	Brazil	120	Phone calls after hospital discharge to educate and evaluate signs of decompensation of HF.	3 months	Standard care	Intervention: 22
Domingues *et al*., 2011 (23)	Brazil	120	Phone calls after hospital discharge to educate and evaluate signs of decompensation of HF.	3 months	Standard care	Control: 42
Domingues *et al*., 2011 (23)	Brazil	120	Phone calls after hospital discharge to educate and evaluate signs of decompensation of HF.	3 months	Standard care	Days of hospital stay at 12 months+
Domingues *et al*., 2011 (23)	Brazil	120	Phone calls after hospital discharge to educate and evaluate signs of decompensation of HF.	3 months	Standard care	Intervention: 4.1 (6.4)
Domingues *et al*., 2011 (23)	Brazil	120	Phone calls after hospital discharge to educate and evaluate signs of decompensation of HF.	3 months	Standard care	Control: 7.6 (12.1)
Doughty *et al*., 2002 (24)	New Zealand	197	Educational brochure on HF and its management. Home visits.	12 months	Standard care	Readmissions* at 12 months
Doughty *et al*., 2002 (24)-	New Zealand	197	Educational brochure on HF and its management. Home visits.	12 months	Standard care	Intervention: 36
Doughty *et al*., 2002 (24)	New Zealand	197	Educational brochure on HF and its management. Home visits.	12 months	Standard care	Control: 65
Dracup *et al*., 2014 (25)	United States	614	Face-to-face education session delivered by a nurse focusing on self-care. Phone calls.	3, 12 and 24 months	Standard care	Readmissions* at 24 months
Dracup *et al*., 2014 (25)	United States	614	Face-to-face education session delivered by a nurse focusing on self-care. Phone calls.	3, 12 and 24 months	Standard care	Intervention: 63
Dracup *et al*., 2014 (25)	United States	614	Face-to-face education session delivered by a nurse focusing on self-care. Phone calls.	3, 12 and 24 months	Standard care	Control: 64
Ducharme *et al.*, 2005 (26)	Canada	230	Visits to the HF clinic to provide education in the management of the disease. Phone calls every month. Educational brochure.	6 months	Standard care	Readmissions* at 6 months
Ducharme *et al.*, 2005 (26)	Canada	230	Visits to the HF clinic to provide education in the management of the disease. Phone calls every month. Educational brochure.	6 months	Standard care	Intervention: 45
Ducharme *et al.*, 2005 (26)	Canada	230	Visits to the HF clinic to provide education in the management of the disease. Phone calls every month. Educational brochure.	6 months	Standard care	Control: 66
Gámez-López *et al*., 2012 (27)	Spain	208	Follow-up in the HF clinic after discharge. Phone call after discharge to reinforce education. Home visit.	12 months	Standard care	Readmissions* at 12 months
Gámez-López *et al*., 2012 (27)	Spain	208	Follow-up in the HF clinic after discharge. Phone call after discharge to reinforce education. Home visit.	12 months	Standard care	Intervention: 11
Gámez-López *et al*., 2012 (27)	Spain	208	Follow-up in the HF clinic after discharge. Phone call after discharge to reinforce education. Home visit.	12 months	Standard care	Control: 14
Gámez-López *et al*., 2012 (27)	Spain	208	Follow-up in the HF clinic after discharge. Phone call after discharge to reinforce education. Home visit.	12 months	Standard care	Days of hospital stay at 12 months+
Gámez-López *et al*., 2012 (27)	Spain	208	Follow-up in the HF clinic after discharge. Phone call after discharge to reinforce education. Home visit.	12 months	Standard care	Intervention: 6.7 (13.5)
Gámez-López *et al*., 2012 (27)	Spain	208	Follow-up in the HF clinic after discharge. Phone call after discharge to reinforce education. Home visit.	12 months	Standard care	Control: 10.7 (22.2)
González-Guerrero *et al.*, 2014 (28)	Spain	116	Flyer with information about the disease. Follow-up call within 48 hours. Reinforcement of education after 10 days. Visits to the geriatric center to reinforce education.	12 months	Standard care	Readmissions* at 12 months
González-Guerrero *et al.*, 2014 (28)	Spain	116	Flyer with information about the disease. Follow-up call within 48 hours. Reinforcement of education after 10 days. Visits to the geriatric center to reinforce education.	12 months	Standard care	Intervention: 14
González-Guerrero *et al.*, 2014 (28)	Spain	116	Flyer with information about the disease. Follow-up call within 48 hours. Reinforcement of education after 10 days. Visits to the geriatric center to reinforce education.	12 months	Standard care	Control: 18
González-Guerrero *et al.*, 2014 (28)	Spain	116	Flyer with information about the disease. Follow-up call within 48 hours. Reinforcement of education after 10 days. Visits to the geriatric center to reinforce education.	12 months	Standard care	Days of hospital stay at 12 months+
González-Guerrero *et al.*, 2014 (28)	Spain	116	Flyer with information about the disease. Follow-up call within 48 hours. Reinforcement of education after 10 days. Visits to the geriatric center to reinforce education.	12 months	Standard care	Intervention: 16.8 (18.2)
González-Guerrero *et al.*, 2014 (28)	Spain	116	Flyer with information about the disease. Follow-up call within 48 hours. Reinforcement of education after 10 days. Visits to the geriatric center to reinforce education.	12 months	Standard care	Control: 20.6 (23.5)
Hägglund *et al.,* 2015 (29)	Sweden	72	Educational sessions at home through a tablet about HF and its management.	3 months	Standard care	Readmissions* at 3 months
Hägglund *et al.,* 2015 (29)	Sweden	72	Educational sessions at home through a tablet about HF and its management.	3 months	Standard care	Intervention: 7
Hägglund *et al.,* 2015 (29)	Sweden	72	Educational sessions at home through a tablet about HF and its management.	3 months	Standard care	Control: 11
Holland *et al*., 2007 (30)	United Kingdom	399	Home visit after discharge to educate on HF and its management. Follow-up visit to reinforce education.	3 and 6 months	Standard care	Readmissions* at 3 months
Holland *et al*., 2007 (30)	United Kingdom	399	Home visit after discharge to educate on HF and its management. Follow-up visit to reinforce education.	3 and 6 months	Standard care	Intervention: 12
Holland *et al*., 2007 (30)	United Kingdom	399	Home visit after discharge to educate on HF and its management. Follow-up visit to reinforce education.	3 and 6 months	Standard care	Control: 9
Holland *et al*., 2007 (30)	United Kingdom	399	Home visit after discharge to educate on HF and its management. Follow-up visit to reinforce education.	3 and 6 months	Standard care	Readmissions* at 6 months
Holland *et al*., 2007 (30)	United Kingdom	399	Home visit after discharge to educate on HF and its management. Follow-up visit to reinforce education.	3 and 6 months	Standard care	Intervention: 1
Holland *et al*., 2007 (30)	United Kingdom	399	Home visit after discharge to educate on HF and its management. Follow-up visit to reinforce education.	3 and 6 months	Standard care	Control: 1
Jaarsma *et al*., 1999 (31)	Netherlands	174	Education about HF, treatment and management during hospitalization. Phone call and home visit.	1, 3 and 9 months	Standard care	Readmissions* at 3 months
Jaarsma *et al*., 1999 (31)	Netherlands	174	Education about HF, treatment and management during hospitalization. Phone call and home visit.	1, 3 and 9 months	Standard care	Intervention: 18
Jaarsma *et al*., 1999 (31)	Netherlands	174	Education about HF, treatment and management during hospitalization. Phone call and home visit.	1, 3 and 9 months	Standard care	Control: 23
Jaarsma *et al*., 1999 (31)	Netherlands	174	Education about HF, treatment and management during hospitalization. Phone call and home visit.	1, 3 and 9 months	Standard care	Days of hospital stay at 3 months+
Jaarsma *et al*., 1999 (31)	Netherlands	174	Education about HF, treatment and management during hospitalization. Phone call and home visit.	1, 3 and 9 months	Standard care	Intervention: 3 (7)
Jaarsma *et al*., 1999 (31)	Netherlands	174	Education about HF, treatment and management during hospitalization. Phone call and home visit.	1, 3 and 9 months	Standard care	Control: 4.1 (10)
Jaarsma *et al*., 2011 (32)	Netherlands	1049	Home visit after discharge and every 6 months to receive education on HF. Additional home visits (basic group). Monthly contact with the nurse, additional visits and telephone follow-up (intensive group).	18 months	Standard care	Readmissions* at 18 months
Jaarsma *et al*., 2011 (32)	Netherlands	1049	Home visit after discharge and every 6 months to receive education on HF. Additional home visits (basic group). Monthly contact with the nurse, additional visits and telephone follow-up (intensive group).	18 months	Standard care	Intervention: 134
Jaarsma *et al*., 2011 (32)	Netherlands	1049	Home visit after discharge and every 6 months to receive education on HF. Additional home visits (basic group). Monthly contact with the nurse, additional visits and telephone follow-up (intensive group).	18 months	Standard care	Control: 120
Jerant *et al.,* 2001 (33)	United States	37	Two home visits after discharge. Phone calls. Telecare.	6 months	Standard care	Readmissions* at 6 months
Jerant *et al.,* 2001 (33)	United States	37	Two home visits after discharge. Phone calls. Telecare.	6 months	Standard care	Intervention: 1
Jerant *et al.,* 2001 (33)	United States	37	Two home visits after discharge. Phone calls. Telecare.	6 months	Standard care	Control: 4
Kato *et al*., 2016 (34)	Japan	38	Education and advice on knowledge about HF and self-care.	24 months	Standard care	Readmissions* at 24 months
Kato *et al*., 2016 (34)	Japan	38	Education and advice on knowledge about HF and self-care.	24 months	Standard care	Intervention: 2
Kato *et al*., 2016 (34)	Japan	38	Education and advice on knowledge about HF and self-care.	24 months	Standard care	Control: 7
Kimmelstiel *et al*., 2004 (35)	United States	200	Home visit. Manual with information on HF.	3 and 6 months	Standard care	Readmissions* at 3 months
Kimmelstiel *et al*., 2004 (35)	United States	200	Home visit. Manual with information on HF.	3 and 6 months	Standard care	Intervention: 15
Kimmelstiel *et al*., 2004 (35)	United States	200	Home visit. Manual with information on HF.	3 and 6 months	Standard care	Control: 24
Kimmelstiel *et al*., 2004 (35)	United States	200	Home visit. Manual with information on HF.	3 and 6 months	Standard care	Days of hospital stay at months+
Kimmelstiel *et al*., 2004 (35)	United States	200	Home visit. Manual with information on HF.	3 and 6 months	Standard care	Intervention: 4.3 (10.2)
Kimmelstiel *et al*., 2004 (35)	United States	200	Home visit. Manual with information on HF.	3 and 6 months	Standard care	Control: 7.8 (19.7)
Koelling *et al*., 2005 (36)	United States	223	Education prior to discharge on the management of HF. Information brochure. Application of self-care questionnaires.	1, 3 and 6 months	Standard care	Readmissions* at 6 months
Koelling *et al*., 2005 (36)	United States	223	Education prior to discharge on the management of HF. Information brochure. Application of self-care questionnaires.	1, 3 and 6 months	Standard care	Intervention: 16
Koelling *et al*., 2005 (36)	United States	223	Education prior to discharge on the management of HF. Information brochure. Application of self-care questionnaires.	1, 3 and 6 months	Standard care	Control: 33
Koelling *et al*., 2005 (36)	United States	223	Education prior to discharge on the management of HF. Information brochure. Application of self-care questionnaires.	1, 3 and 6 months	Standard care	Days of hospital stay at 6 months+
Koelling *et al*., 2005 (36)	United States	223	Education prior to discharge on the management of HF. Information brochure. Application of self-care questionnaires.	1, 3 and 6 months	Standard care	Intervention: 13.1 (36)
Koelling *et al*., 2005 (3^6^)	United States	223	Education prior to discharge on the management of HF. Information brochure. Application of self-care questionnaires.	1, 3 and 6 months	Standard care	Control: 17.1 (37)
Krumholz *et al.*, 2002 (37)	United States	88	Sequential education on HF and its management. Educational brochure. Home visits. Telemonitoring to reinforce education.	12 months	Standard care	Readmissions* at 12 months
Krumholz *et al.*, 2002 (37)	United States	88	Sequential education on HF and its management. Educational brochure. Home visits. Telemonitoring to reinforce education.	12 months	Standard care	Intervention: 22
Krumholz *et al.*, 2002 (37)	United States	88	Sequential education on HF and its management. Educational brochure. Home visits. Telemonitoring to reinforce education.	12 months	Standard care	Control: 42
Krumholz *et al.*, 2002 (37)	United States	88	Sequential education on HF and its management. Educational brochure. Home visits. Telemonitoring to reinforce education.	12 months	Standard care	Days of hospital stay at 12 months+
Krumholz *et al.*, 2002 (37)	United States	88	Sequential education on HF and its management. Educational brochure. Home visits. Telemonitoring to reinforce education.	12 months	Standard care	Intervention: 4.1 (6.4)
Krumholz *et al.*, 2002 (37)	United States	88	Sequential education on HF and its management. Educational brochure. Home visits. Telemonitoring to reinforce education.	12 months	Standard care	Control: 7.6 (12.1)
Leventhal *et al.,* 2011 (38)	Switzerland	42	Home visit to provide HF education. Phone calls to reinforce education. Educational kit with self-care procedures.	3, 6, 9 and 12 months	Standard care	Readmissions* at 12 months
Leventhal *et al.,* 2011 (38)	Switzerland	42	Home visit to provide HF education. Phone calls to reinforce education. Educational kit with self-care procedures.	3, 6, 9 and 12 months	Standard care	Intervention: 1
Leventhal *et al.,* 2011 (38)	Switzerland	42	Home visit to provide HF education. Phone calls to reinforce education. Educational kit with self-care procedures.	3, 6, 9 and 12 months	Standard care	Control: 2
Mau *et al*., 2017 (39)	United States	150	Educational modules on HF and its treatment.	12 months	Standard care	Readmissions* at 12 months
Mau *et al*., 2017 (39)	United States	150	Educational modules on HF and its treatment.	12 months	Standard care	Intervention: 5
Mau *et al*., 2017 (39)	United States	150	Educational modules on HF and its treatment.	12 months	Standard care	Control: 18
Melin *et al*., 2018 (40)	Sweden	72	Education of self-care practices and management of HF.	6 months	Standard care	Readmissions* at 6 months
Melin *et al*., 2018 (40)	Sweden	72	Education of self-care practices and management of HF.	6 months	Standard care	Intervention: 14
Melin *et al*., 2018 (40)	Sweden	72	Education of self-care practices and management of HF.	6 months	Standard care	Control: 16
Naylor *et al*., 2004 (41)	United States	239	Daily education during the hospitalization period. Home visits to reinforce education about HF and its management.	3, 6 and 12 months - 2, 6, 12, 26, 52 weeks	Standard care	Readmissions* at 12 months
Naylor *et al*., 2004 (41)	United States	239	Daily education during the hospitalization period. Home visits to reinforce education about HF and its management.	3, 6 and 12 months - 2, 6, 12, 26, 52 weeks	Standard care	Intervention: 40
Naylor *et al*., 2004 (41)	United States	239	Daily education during the hospitalization period. Home visits to reinforce education about HF and its management.	3, 6 and 12 months - 2, 6, 12, 26, 52 weeks	Standard care	Control: 72
Naylor *et al*., 2004 (41)	United States	239	Daily education during the hospitalization period. Home visits to reinforce education about HF and its management.	3, 6 and 12 months - 2, 6, 12, 26, 52 weeks	Standard care	Days of hospital stay at 12 months+
Naylor *et al*., 2004 (41)	United States	239	Daily education during the hospitalization period. Home visits to reinforce education about HF and its management.	3, 6 and 12 months - 2, 6, 12, 26, 52 weeks	Standard care	Intervention: 11.1 (7.2)
Naylor *et al*., 2004 (41)	United States	239	Daily education during the hospitalization period. Home visits to reinforce education about HF and its management.	3, 6 and 12 months - 2, 6, 12, 26, 52 weeks	Standard care	Control: 14.5 (13.4)
Negarandeh *et al*., 2019 (42)	Iran	80	Telemonitoring with HF education.	1 and 3 months	Standard care	Readmissions* at 3 months
Negarandeh *et al*., 2019 (42)	Iran	80	Telemonitoring with HF education.	1 and 3 months	Standard care	Intervention: 7
Negarandeh *et al*., 2019 (42)	Iran	80	Telemonitoring with HF education.	1 and 3 months	Standard care	Control: 14
Otsu *et al*., 2011 (43)	Japan	102	Educational program in HF clinic about the disease and its management.	3, 6, 9 and 12 months	Standard care	Readmissions* at 6 months
Otsu *et al*., 2011 (43)	Japan	102	Educational program in HF clinic about the disease and its management.	3, 6, 9 and 12 months	Standard care	Intervention: 1
Otsu *et al*., 2011 (43)	Japan	102	Educational program in HF clinic about the disease and its management.	3, 6, 9 and 12 months	Standard care	Control: 1
Ramachandran *et al.,* 2007 (44)	India	50	Education on HF, management and treatment. Reinforcement of education by phone calls. Patient education manual. Follow-up in the HF clinic.	6 months	Standard care	Readmissions* at 6 months
Ramachandran *et al.,* 2007 (44)	India	50	Education on HF, management and treatment. Reinforcement of education by phone calls. Patient education manual. Follow-up in the HF clinic.	6 months	Standard care	Intervention: 6
Ramachandran *et al.,* 2007 (44)	India	50	Education on HF, management and treatment. Reinforcement of education by phone calls. Patient education manual. Follow-up in the HF clinic.	6 months	Standard care	Control: 4
Rodríguez-Gázquez *et al.,* 2012 (45)	Colombia	63	Educational program in nursing (educational meetings, home visits, telenursing and a printed book) in the improvement of self-care behaviors.	9 months	Standard care	Readmissions* at 9 months
Rodríguez-Gázquez *et al.,* 2012 (45)	Colombia	63	Educational program in nursing (educational meetings, home visits, telenursing and a printed book) in the improvement of self-care behaviors.	9 months	Standard care	Intervention: 30
Rodríguez-Gázquez *et al.,* 2012 (45)	Colombia	63	Educational program in nursing (educational meetings, home visits, telenursing and a printed book) in the improvement of self-care behaviors.	9 months	Standard care	Control: 24
Ruschel *et al*., 2018 (46)	Brazil	252	Home visits and phone calls. Education on HF and self-care practices.	6 months	Standard care	Readmissions* at 6 months
Ruschel *et al*., 2018 (46)	Brazil	252	Home visits and phone calls. Education on HF and self-care practices.	6 months	Standard care	Intervention: 30
Ruschel *et al*., 2018 (46)	Brazil	252	Home visits and phone calls. Education on HF and self-care practices.	6 months	Standard care	Control: 30
Sethares *et al*., 2004 (47)	United States	70	Education about HF during hospitalization. Reinforcement education after discharge.	3 months	Standard care	Readmissions* at 3 months
Sethares *et al*., 2004 (47)	United States	70	Education about HF during hospitalization. Reinforcement education after discharge.	3 months	Standard care	Intervention: 6
Sethares *et al*., 2004 (47)	United States	70	Education about HF during hospitalization. Reinforcement education after discharge.	3 months	Standard care	Control: 12
Stewart *et al*., 2015 (48)	Australia and New Zealand	624	Home visit after discharge. Education on HF and its management. Personalized care plan.	1 and 36 months	Standard care	Readmissions* at 36 months
Stewart *et al*., 2015 (48)	Australia and New Zealand	624	Home visit after discharge. Education on HF and its management. Personalized care plan.	1 and 36 months	Standard care	Intervention: 17
Stewart *et al*., 2015 (48)	Australia and New Zealand	624	Home visit after discharge. Education on HF and its management. Personalized care plan.	1 and 36 months	Standard care	Control: 17
Tomita *et al*., 2009 (49)	United States	40	Information online about HF and its management.	6 and 12 months	Standard care	Days of hospital stay at 6 months+
Tomita *et al*., 2009 (49)	United States	40	Information online about HF and its management.	6 and 12 months	Standard care	Intervention: 1 (2.45)
Tomita *et al*., 2009 (49)	United States	40	Information online about HF and its management.	6 and 12 months	Standard care	Control: 0.84 (1.89)
Tomita *et al*., 2009 (49)	United States	40	Information online about HF and its management.	6 and 12 months	Standard care	Days of hospital stay at 12 months+
Tomita *et al*., 2009 (49)	United States	40	Information online about HF and its management.	6 and 12 months	Standard care	Intervention: 1.23 (2.55)
Tomita *et al*., 2009 (49)	United States	40	Information online about HF and its management.	6 and 12 months	Standard care	Control: 2.42 (5.07)
Tsuchihashi‐Makaya *et al*., 2013 (50)	Japan	164	Pre-discharge education on HF and its management. Educational brochure. Home visits once a week for two months. Monthly telephone follow-up for six months.	2, 6 and 12 months	Standard care	Readmissions* at 6 months
Tsuchihashi‐Makaya *et al*., 2013 (50)	Japan	164	Pre-discharge education on HF and its management. Educational brochure. Home visits once a week for two months. Monthly telephone follow-up for six months.	2, 6 and 12 months	Standard care	Intervention: 6
Tsuchihashi‐Makaya *et al*., 2013 (50)	Japan	164	Pre-discharge education on HF and its management. Educational brochure. Home visits once a week for two months. Monthly telephone follow-up for six months.	2, 6 and 12 months	Standard care	Control: 15
Tsuchihashi‐Makaya *et al*., 2013 (50)	Japan	164	Pre-discharge education on HF and its management. Educational brochure. Home visits once a week for two months. Monthly telephone follow-up for six months.	2, 6 and 12 months	Standard care	Readmissions* at 12 months
Tsuchihashi‐Makaya *et al*., 2013 (50)	Japan	164	Pre-discharge education on HF and its management. Educational brochure. Home visits once a week for two months. Monthly telephone follow-up for six months.	2, 6 and 12 months	Standard care	Intervention: 6
Tsuchihashi‐Makaya *et al*., 2013 (50)	Japan	164	Pre-discharge education on HF and its management. Educational brochure. Home visits once a week for two months. Monthly telephone follow-up for six months.	2, 6 and 12 months	Standard care	Control: 9
Wakefield *et al.*, 2008 (51)	United States	148	Follow-up after discharge. Phone calls to provide HF education.	3, 6 and 12 months	Standard care	Readmissions* at 12 months
Wakefield *et al.*, 2008 (51)	United States	148	Follow-up after discharge. Phone calls to provide HF education.	3, 6 and 12 months	Standard care	Intervention: 21
Wakefield *et al.*, 2008 (51)	United States	148	Follow-up after discharge. Phone calls to provide HF education.	3, 6 and 12 months	Standard care	Control: 29
Wierzchowiecki *et al*., 2006 (52)	Poland	160	Education and follow-up in the HF clinic. Phone calls for educational reinforcement.	12 months	Standard care	Readmissions* at 12 months
Wierzchowiecki *et al*., 2006 (52)	Poland	160	Education and follow-up in the HF clinic. Phone calls for educational reinforcement.	12 months	Standard care	Intervention: 13
Wierzchowiecki *et al*., 2006 (52)	Poland	160	Education and follow-up in the HF clinic. Phone calls for educational reinforcement.	12 months	Standard care	Control: 25
Wright *et al*., 2003 (53)	New Zealand	197	Clinical review after discharge. Home visits every 6 weeks to educate on HF, treatment and management.	12 months	Standard care	Readmissions* at 12 months
Wright *et al*., 2003 (53)	New Zealand	197	Clinical review after discharge. Home visits every 6 weeks to educate on HF, treatment and management.	12 months	Standard care	Intervention: 46
Wright *et al*., 2003 (53)	New Zealand	197	Clinical review after discharge. Home visits every 6 weeks to educate on HF, treatment and management.	12 months	Standard care	Control: 18
Wright *et al*., 2003 (53)	New Zealand	197	Clinical review after discharge. Home visits every 6 weeks to educate on HF, treatment and management.	12 months	Standard care	Days of hospital stay at 12 months+
Wright *et al*., 2003 (53)	New Zealand	197	Clinical review after discharge. Home visits every 6 weeks to educate on HF, treatment and management.	12 months	Standard care	Intervention: 9.4 (13.6)
Wright *et al*., 2003 (53)	New Zealand	197	Clinical review after discharge. Home visits every 6 weeks to educate on HF, treatment and management.	12 months	Standard care	Control: 14.9 (18.8)
Yu *et al*., 2015 (54)	China	178	Education before discharge about HF. Home visits and phone calls for educational reinforcement.	6 weeks, 3 and 9 months	Standard care	Readmissions* at 6 weeks - 3 months - 9 months
Yu *et al*., 2015 (54)	China	178	Education before discharge about HF. Home visits and phone calls for educational reinforcement.	6 weeks, 3 and 9 months	Standard care	Intervention: 7 - 12 - 6
Yu *et al*., 2015 (54)	China	178	Education before discharge about HF. Home visits and phone calls for educational reinforcement.	6 weeks, 3 and 9 months	Standard care	Control: 10 - 7 - 3

HF: heart failure; * Data presented as number of patients readmitted due to decompensation of HF; ^+^ Data presented as mean (standard deviation).

Table 1 shows that this SR included 9688 adult patients with HF. The studies were published between 1999 and 2019. The investigations were conducted in 16 countries, with the highest number of these in the United States and Spain (16 and 5, respectively). The follow-up of the studies included was carried out during different periods, comprised between the first month after the intervention and at 36 months. Studies with follow-up at 3, 6 and 12 months were predominant.

With respect to the educational interventions, these were diverse; however, common strategies were found in the studies included, like: education during hospitalization, telephone follow-up, home visits to reinforce the education, visits to HF clinics, and delivery of printed or digital educational material (brochures, videos or manuals) for consultation by the patients. The education centered on knowledge of the disease, warning signs, diet, and self-care practices.

Regarding the comparison with the control group, it was found that in general, the usual care was perceived as the clinical care by the cardiologist and a single control visit in the outpatient care service.

### Analysis of the risk of bias of the studies included

The evaluation of the risk of bias of the studies is presented in Table 2. According with the parameters evaluated by the RoB 1 tool,(9) it was obtained that all the studies performed an adequate random sequence generation; allocation concealment was optimal in 65.1% of the studies included. Due to the nature of the educational interventions, in the studies it was not possible to conduct blinding of the patients and of the staff who offered the interventions. In relation blinding of outcome assessment, only 48.8% low risk was presented for this domain. In all, 93% of the studies described clearly the losses presented during the follow-up and if the data analysis was carried out through intention of treatment, which reduced the risk of bias due to incomplete results. Finally, regarding the risk of selective reporting of the results, it was found that 97.7% described the results proposed since the beginning (Table 2).

**Table 2:** Assessment of risk of bias among included studies

**Studies**	Random sequence generation	Allocation concealment	Blinding of participants and personnel	Blinding of outcome assessment	Incomplete outcome data	Selective reporting
Aldamiz-Echevarría *et al.,* 2007 (10)	Low risk	Low risk	Low risk	Low risk	Low risk	Low risk
Atienza *et al*., 2004 (11)	Low risk	Low risk	Low risk	Unclear risk	Low risk	Low risk
Blue *et al*., 2001 (12)	Low risk	Low risk	Low risk	Unclear risk	Low risk	Low risk
Boyde *et al.,* 2018 (13)	Low risk	Low risk	Low risk	High risk	Low risk	Low risk
Brian *et al.,* 2009 (14)	Low risk	Low risk	Low risk	Low risk	Low risk	Low risk
Brotons *et al*., 2009 (15)	Low risk	Low risk	Low risk	Low risk	Low risk	Low risk
Cañon-Montañez *et al*., 2013 (16)	Low risk	Low risk	Low risk	Low risk	Low risk	Low risk
Cui *et al*., 2019 (17)	Low risk	Low risk	Low risk	Low risk	Low risk	Low risk
Davis *et al*., 2012 (18)	Low risk	Unclear risk	Low risk	Low risk	Low risk	Low risk
De Souza *et al.*, 2014 (19)	Low risk	Low risk	Low risk	Low risk	Low risk	Low risk
DeBusk *et al*., 2004 (20)	Low risk	Low risk	Low risk	Low risk	Low risk	Low risk
Delaney *et al*., 2013 (21)	Low risk	Low risk	Low risk	Unclear risk	Low risk	Low risk
Dewalt *et al.,* 2006 (22)	Low risk	Low risk	Low risk	High risk	Low risk	Low risk
Domingues *et al*., 2011 (23)	Low risk	Unclear risk	Low risk	Unclear risk	Unclear risk	Low risk
Doughty *et al*., 2002 (24)	Low risk	Low risk	Low risk	Low risk	Low risk	Low risk
Dracup *et al*., 2014 (25)	Low risk	Low risk	Low risk	Low risk	Low risk	Low risk
Ducharme *et al.,* 2005 (26)	Low risk	Low risk	Low risk	Low risk	Low risk	Low risk
Gámez-López *et al.,* 2012 (27)	Low risk	Unclear risk	Low risk	Low risk	Unclear risk	Low risk
González-Guerrero *et al*., 2014 (28)	Low risk	Low risk	Low risk	Unclear risk	Low risk	Low risk
Hägglund *et al*., 2015 (29)	Low risk	Unclear risk	Low risk	Unclear risk	Low risk	Low risk
Holland *et al.,* 2007 (30)	Low risk	Low risk	Low risk	Unclear risk	Low risk	Low risk
Jaarsma *et al.,* 1999 (31)	Low risk	Low risk	Low risk	Low risk	Low risk	Low risk
Jaarsma *et al.,* 2011 (32)	Low risk	Low risk	Low risk	Unclear risk	Low risk	Low risk
Jerant *et al.,* 2001(33)	Low risk	Low risk	Low risk	Unclear risk	Low risk	Low risk
Kato *et al.,* 2016 (34)	Low risk	Low risk	Low risk	Low risk	Low risk	Low risk
Kimmelstiel *et al*., 2004 (35)	Low risk	High risk	Low risk	Low risk	Low risk	Low risk
Koelling *et al*., 2005 (36)	Low risk	Low risk	Low risk	Low risk	Low risk	Low risk
Krumholz *et al.,* 2002 (37)	Low risk	Unclear risk	Low risk	Unclear risk	Low risk	Unclear risk
Leventhal *et al.,* 2011 (38)	Low risk	Low risk	Low risk	Low risk	Low risk	Low risk
Mau *et al*., 2017 (39)	Low risk	High risk	Low risk	Unclear risk	Low risk	Low risk
Melin *et al*., 2018 (40)	Low risk	High risk	Low risk	Low risk	Low risk	Low risk
Naylor *et a*l., 2004 (41)	Low risk	Low risk	Low risk	Low risk	Low risk	Low risk
Negarandeh *et al*., 2019 (42)	Low risk	Low risk	Low risk	Unclear risk	Low risk	Low risk
Otsu *et al*., 2011 (43)	Low risk	Unclear risk	Low risk	High risk	Low risk	Low risk
Ramachandran *et al*., 2007 (44)	Low risk	Low risk	Low risk	High risk	Low risk	Low risk
Rodríguez-Gázquez *et al.,* 2012 (45)	Low risk	Low risk	Low risk	Unclear risk	Low risk	Low risk
Ruschel *et al*., 2018 (46)	Low risk	Unclear risk	Low risk	Low risk	Low risk	Low risk
Sethares *et al*., 2004 (47)	Low risk	Low risk	Low risk	Low risk	Low risk	Low risk
Stewart *et al.,* 2015 (48)	Low risk	Low risk	Low risk	Low risk	Low risk	Low risk
Tomita *et al*., 2009 (49)	Low risk	Unclear risk	Low risk	Unclear risk	Low risk	Low risk
Tsuchihashi‐Makaya *et al*. 2013 (50)	Low risk	Unclear risk	Low risk	Unclear risk	Low risk	Low risk
Walkefield *et al*., 2008 (51)	Low risk	Low risk	Low risk	Unclear risk	Low risk	Low risk
Wierzchowiecki *et al*., 2006 (52)	Low risk	Unclear risk	Low risk	Unclear risk	Unclear risk	Low risk
Wright *et al*., 2003 (53)	Low risk	Unclear risk	Low risk	Unclear risk	Low risk	Low risk
Yu *et al*., 2015 (54)	Low risk	Low risk	Low risk	Unclear risk	Low risk	Low risk

### Meta-analysis

**Figure 2:**
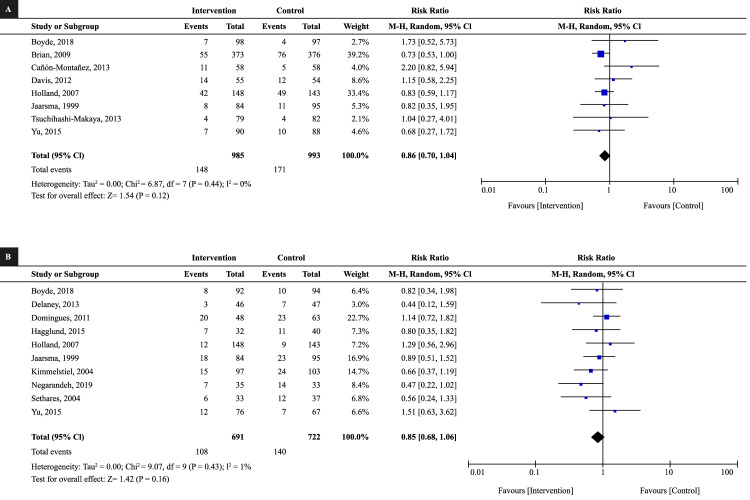
Meta-analysis of the effect of the educational interventions on reducing readmissions due to heart failure. (A) Follow-up <3 months, (B) Follow-up at 3 months

The work included the results from 43 studies and analyzed hospital readmissions, during different follow-up periods, *i.e*., 6 weeks, 1 month, 2, 3, 6, 9, 12, and 24 months. Upon evaluating the combined effect, no statistically significant results were obtained in studies with follow-up <3 months nor at three months (Figure 2). Significant results were also not found at nine months (RR: 0.98, 95% CI: 0.64 to 1.54, I^2^: 61%), as well as at 24 months (RR: 0.72, 95% CI: 0.24 to 2.17, I^2^: 62%).

**Figure 3:**
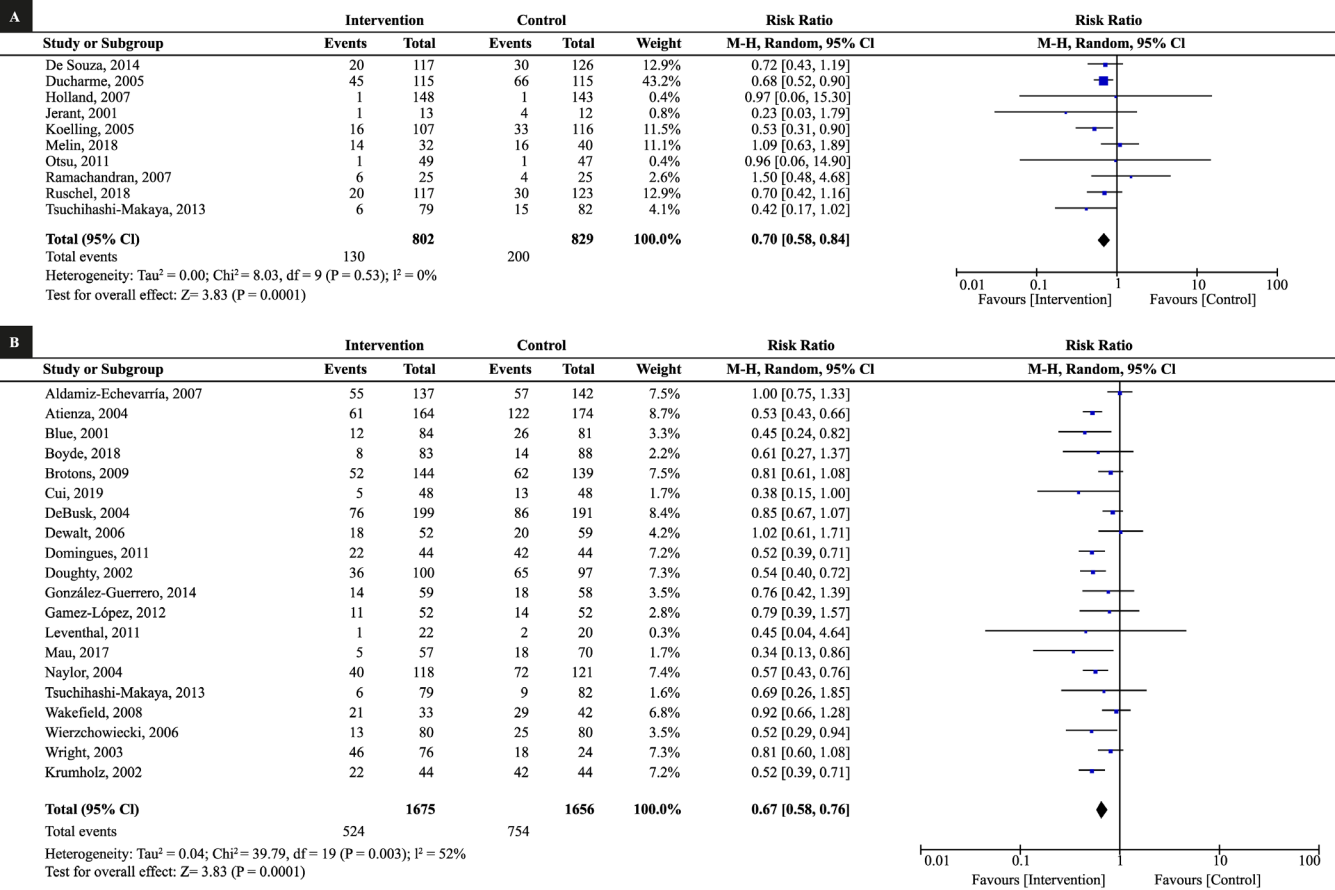
Meta-analysis of the effect of educational interventions on reducing readmissions due to heart failure. (A) Follow-up at 6 months, (B) Follow-up at 12 months

The MA of studies with follow-up at six months showed a 30% decrease in readmissions (RR: 0.70; 95% CI: 0.58 to 0.84; I^2^: 0%) and the 12-month follow-up evidenced 33% reduction (RR: 0.67; 95% CI: 0.58 to 0.76; I^2^: 52%); both analyses in favor of the group of educational interventions (Figure 3). 

**Figure 4:**
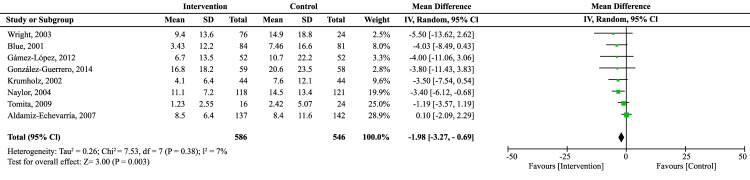
Meta-analysis of the effect of educational interventions on reducing days of hospital stay due to heart failure at 12 months of follow-up

For the secondary outcome, days of hospital stay, no favorable effect was found of the educational interventions during the follow-up at three months (MD: -1.71; 95% CI: -3.87 to -0.46; I^2^: 0%) and six months (MD: 0.07; 95% CI: -1.33 to 1.45; I^2^: 0%). Nevertheless, the MA with follow-up at 12 months (Figure 4) evidenced a reduction of approximately two days in patients who received the educational interventions (MD: -1.98; 95% CI: -3.27 to -0.69; I^2^: 7%).

### Evaluation of publication bias or bias due to missing results

**Figure 5:**
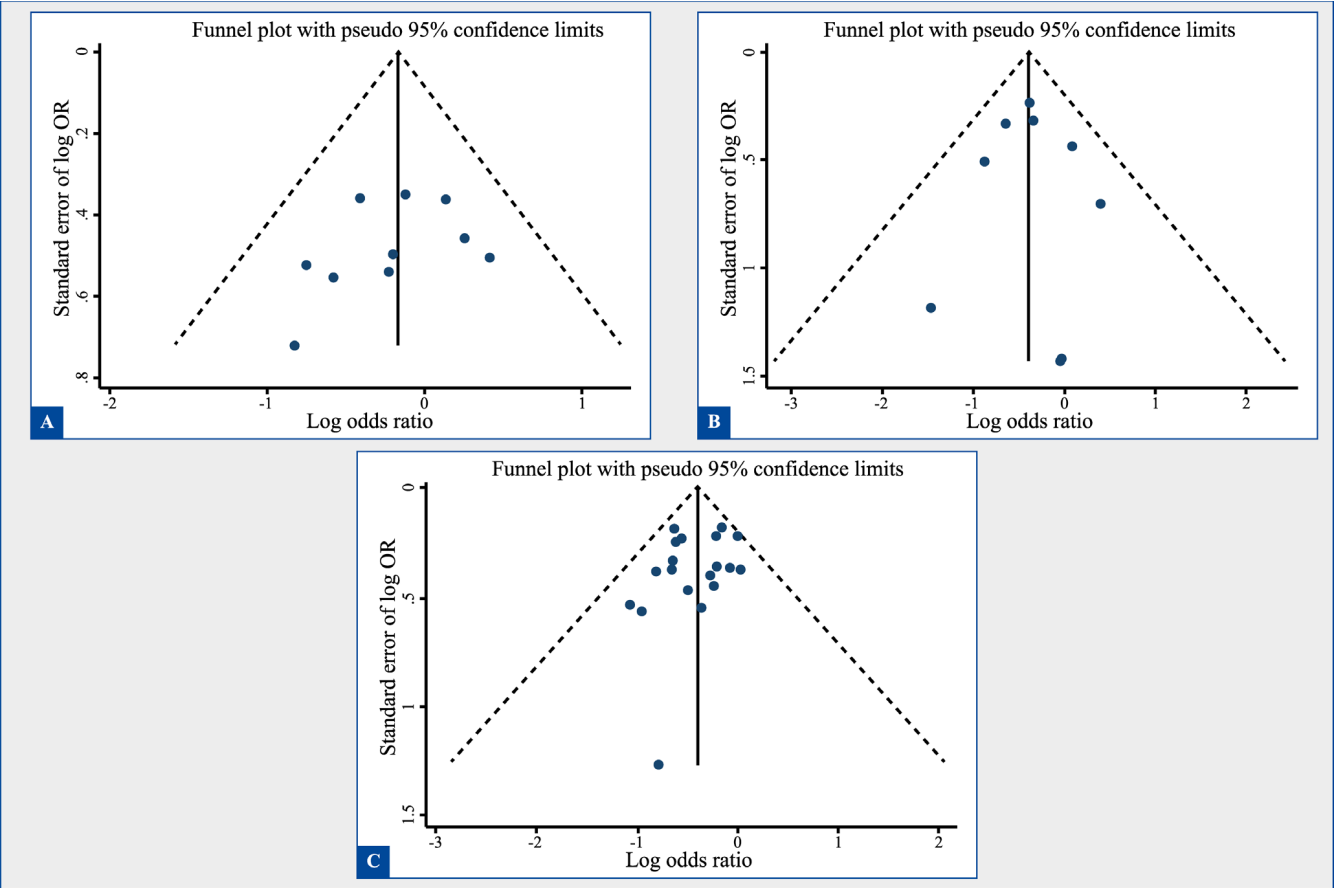
Funnel plot to analyze publication bias or bias due to missing results during three follow-up times. (A) 3 months, (B) 6 months, (C) 12 months

Figure 5 shows funnel plot graphics to evaluate publication bias under analysis of 10 or more studies (three months, six months, and twelve months of follow-up). For the three times of follow-up, it is possible to observe generally a funnel shape that indicates that the studies are distributed uniformly on both sides of the average, which suggests lack of publication bias. The Egger statistical test also indicated absence of publication bias (3 months, p = 0.30; 6 months, p = 0.87, and 12 months, p = 0.26).

## Discussion

This up-to-date synthesis of the evidence shows the favorable combined effect of educational interventions during prolonged follow-up times (six and twelve months) to reduce readmissions and time of hospital stay in adults with HF. These results are coherent with other SR and MA conducted prior to this study.(55-57) In addition, the results found reinforce the importance of education for patients and of the multidisciplinary management of the HF syndrome. Similarly, these educational strategies become an alternative of effective intervention to improve the clinical outcomes of patients and which can be useful to reduce costs associated with health services due to HF decompensation. Within this context, a 2017 SR (55) concluded that educational interventions, especially those guided by nurses, have positive effects on decreasing readmissions due to HF.

Two of its studies, which are also part of this SR(38,42) evidenced 50% reduction in readmissions when patients were subjected to educational interventions. In addition, an MA from 2019,(56) that included seven of the RCTs from this study, demonstrates a reduction in hospital readmissions due to HF in follow-up from 6 to 12 months of 27% (RR: 0.73; 95% CI: 0.61 to 0.88; I^2^: 0%) and a general 22% reduction, which groups all the follow-up times. The previously stated, reaffirms the results obtained in this study and gives value to educational interventions as a low-cost strategy to improve the clinical response of patients with HF.

Likewise, another MA from 2019,(57) obtained similar data. The researchers showed reduction of readmissions at 12 months of 36% (RR: 0.64; 95% CI: 0.53 to 0.78; I^2^: 51%). Moreover, this study also evidenced a decrease of approximately two days in hospital stay of adult patients with HF at 12-month follow-up and favorable for the educational interventions. However, no evidence was found of other SR or MA that have evaluated the effect of educational interventions for this result, becoming a significant contribution of this SR and which opens an important path to study this clinical outcome.(57) These results of the evidence can be a starting point to restructure nursing care and management programs for adults with HF. A proactive scenario is proposed in which patients after their discharge continue being a priority and responsibility for health institutions to avoid new readmissions. The findings of studies with prolonged follow-up times show that companionship and active monitoring of patients by a multidisciplinary team generate a positive impact on the clinical outcomes of patients. (56,57)

Another relevant aspect of this SR is that the educational interventions from the studies selected were variables on frequency, duration, methodology and personnel in charge of conducting them. Nevertheless, it is worth highlighting that a vast number of them were carried out by the nursing staff experienced in the cardiovascular area, which reinforces the importance of the nurses’ educator role as an effective strategy in reducing hospital readmissions and maintaining the quality of life of patients with HF. The aforementioned is based on nurses being the professionals called on to provide primary care in patients with chronic diseases.(58,59)

Also, it is important to mention although the study followed the methodological recommendations by the Cochrane Collaboration, this SR and MA had some limitations. First, lack of information is highlighted on the blinding of outcome assessment in some studies. Second, no additional analyses or meta-regressions were performed to explain possible sources of heterogeneity during some follow-up times I^2^ values > 60%. Lastly, this SR and MA did not use the GRADE (Grading of Recommendations, Assessment, Development and Evaluation) methodology to evaluate the degrees of recommendation of the studies selected. Nonetheless, the evaluation of the risk of bias de los RCTs showed that most of the studies included had low risk of bias for the principal domains of the Cochrane RoB 1 tool.

In conclusion, this study demonstrates the protective effect of the educational interventions in adult patients with HF, compared with usual care, to reduce readmissions and days of hospital stay due to decompensation of the disease. Additionally, the results can be useful to reaffirm the need to implement in the clinical practice these intervention strategies during broad follow-up periods and which approach the patient during the transition from hospital to the home. Finally, the importance of participation of nurses in the multidisciplinary teams for the therapeutic approach of adult patients with HF is evident.
